# Translational Research in Audiology

**DOI:** 10.3390/audiolres13050063

**Published:** 2023-09-25

**Authors:** Agnieszka J. Szczepek

**Affiliations:** 1 Department of Otorhinolaryngology, Head and Neck Surgery, Charité-Universitätsmedizin Berlin, Corporate Member of Freie Universität Berlin, Humboldt-Universität zu Berlin, 10117 Berlin, Germany; agnes.szczepek@charite.de; 2 Faculty of Medicine and Health Sciences, University of Zielona Gora, 65-046 Zielona Gora, Poland

The importance of translational research in the medical sciences is growing logarithmically, as this type of research provides the translation of basic research into a clinical product (a drug, therapeutic agent or means of monitoring a disease), as well as the inverse translation of clinical findings into basic research models. This special issue is devoted to translational research in audiology. Unfortunately, the terms “translational audiology” or “translational research in audiology” are still rarely used which encourages a change to increase the visibility of such publications. Among the many benefits associated with using the term “translational audiology” is identifying this type of research as a distinct stream in the science of audiology and emphasizing its practical application in clinical work. How often the term “translational audiology” is used in the literature, what articles have been published using the term, and the rationale and implications of such publications can be found in the review “Translational Research in Audiology: Presence in the Literature” [1].

The translational process often begins in the basic research laboratory. It is essential for clinical success that basic research experiments are reproducible. One of the manuscripts in our special issue addresses this by providing recommendations for performing auditory brainstem response (ABR) in small animals. These recommendations developed by Domarecka et al. are intended to improve the reproducibility of basic audiological research (“Universal Recommendations on Planning and Performing the Auditory Brainstem Responses (ABR) with a Focus on Mice and Rats”) [2].

ABR was the audiometric method used by Blebea et al. to measure the hearing thresholds of rats following mechanical injury to the cochlea when one ear was treated with Pluronic-coated gold nanoparticles containing dexamethasone and the other ear was treated with dexamethasone alone. The comparative analysis of the ABR results demonstrated the benefit of using Pluronic-coated gold nanoparticles over dexamethasone alone (“The Effect of Pluronic-Coated Gold Nanoparticles in Hearing Preservation Following Cochlear Implantation—Pilot Study”) [3]. The investigators suggested that the results of this study could be translated to modify the methods of cochlear implantation in patients with residual hearing, such as those with Meniere’s disease.

A classic example of the translational approach in audiology was proposed by van Zwieten et al. in a paper suggesting the use of deep brain stimulation to treat refractory tinnitus (“A Protocol for Deep Brain Stimulation for Refractory Tinnitus: From Rat Model to Human Pilot Study”) [4]. The health problems associated with tinnitus (e.g., insomnia or difficulty concentrating) can sometimes be extremely distressing, prompting patients and researchers to seek unusual solutions. In order to implement such a solution, the authors developed a translational protocol based on the results of the animal studies.

Tinnitus therapy is the responsibility of many medical disciplines, and many patients seeking help turn to audiologists. However, the education of audiologists varies from country to country and sometimes from one school of audiology to another. In addition, the job title “audiologist” may have various meanings in different settings. This is associated with diverse levels of responsibility and professional obligations. In a communication paper, Marc Fagelson discusses tinnitus-related training for audiologists in the USA (“Tinnitus education for audiologists is a ship at sea: is it coming or going?”) [5].

Implantable hearing aids have changed the lives of many people who have lost their hearing or were born deaf. However, regardless of the effectiveness of the treatment, implantation should not be applied when people are unwilling to be treated or in minors if their guardians do not consent. This issue and related reasons are further discussed by Bendowska et al. in the article “The Ethics of Translational Audiology” which raises awareness about treating the deaf community with dignity and respecting their wishes, which are sometimes at odds with those of hearing people [6].

Sanchez-Lopez et al. provided an excellent example of translational research that focused on translating a hearing aid fitting strategy to the setting needed for large clinical trials (“Towards auditory profile-based hearing-aid fittings: BEAR rationale and clinical implementation”). This group found the first fitting strategy that prescribes gain targets and adjusts advanced hearing aid features for the purpose of clinical research. Other applications could also benefit from using such a fitting strategy [7].

Ryota Shimokura tackled a very interesting subject of autonomous sensory meridian response (ASMR). ASMR is a tingling sensation originating on the scalp and progressing down the spine. Various sound or visual triggers can induce ASMR, and the manuscript “Sound Quality Factors Inducing the Autonomous Sensory Meridian Response” focused on characterizing sound that induces ASMR [8]. The interesting finding of that study was that the human voice could trigger stronger ASMR than the sounds of nature.

The paper by Bassiouni et al., “Lateralization Pattern of the Weber Tuning Fork Test in Longstanding Unilateral Profound Hearing Loss: Implications for Cochlear Implantation” is thought-provoking [9]. It shows that a significant proportion of patients with unilateral hearing loss, despite the unilateral hearing commonly associated with Weber test lateralization, report a lack of such lateralization. Bassiouni suggests that since the duration of deafness correlates positively with this unexpected effect, the loss of Weber lateralization in unilateral deaf patients may result from a central adaptation of sound processing pathway. This finding needs to be considered in cochlear implant candidates. The authors also postulate the need for reverse translational research in this area.

A very interesting topic of the therapeutic implication of low temperature on the outcome of inner ear diseases was put on the spot in a literature review paper, “The Otoprotective Effect of Ear Cryotherapy: Systematic Review and Future Perspectives” [10]. However, the basic research models used whole-body cooling techniques and could not be translated to clinical settings. The general conclusion of the review authors was that the therapeutic use of low temperatures is a promising approach and should be explored further.

There were dark clouds on the horizon in June 2022 when we received the sad news of the untimely and unexpected death of our long-time colleague and wonderful human being—Rev. Prof. David Baguley. David Baguley was active in translational audiology and contributed many publications and chapters to a book on the subject. Don McFerran (ENT surgeon) and Laurence McKenna (clinical psychologist) knew David very well and have taken on the challenge of describing the outline of his life and achievements as a fulfilled audiologist, a beloved Church of England clergyman, an admired academic mentor and, above all, a trusted friend, colleague and family man (“In Memoriam: David Mark Baguley”) [11].

I would like to dedicate this special issue to the memory of Rev. Prof. David Mark Baguley.
